# Immunoproteomics: The Key to Discovery of New Vaccine Antigens Against Bacterial Respiratory Infections

**DOI:** 10.2174/138920312804871184

**Published:** 2012-12

**Authors:** Ruth Dennehy, Siobhán McClean

**Affiliations:** Centre of Microbial Host Interactions, Centre of Applied Science for Health, Institute of Technology Tallaght, Old Blessington Road, Dublin 24, Ireland

**Keywords:** Antigens, immunoproteomics, immunoreactive proteins, respiratory disease, vaccine, virulence.

## Abstract

The increase in antibiotic resistance and the shortage of new antimicrobials to prevent difficult bacterial infections underlines the importance of prophylactic therapies to prevent infection by bacterial pathogens. Vaccination has reduced the incidence of many serious diseases, including respiratory bacterial infections. However, there are many pathogens for which no vaccine is available and some vaccines are not effective among all age groups or among immunocompromised individuals. Immunoproteomics is a powerful technique which has been used to identify potential vaccine candidates to protect against pathogenic bacteria. The combination of proteomics with the detection of immunoreactive antigens using serum highlights immunogenic proteins that are expressed during infection. This is particularly useful when patient serum is used as the antigens that promote a humoral response during human infection are identified. This review outlines examples of vaccine candidates that have been identified using immunoproteomics and have successfully protected animals against challenge when tested in immunisation studies. Many immunoreactive proteins are common to several unrelated pathogens, however some of these are not always protective in animal immunisation and challenge studies. Furthermore, examples of well-established immunogens, including *Bordetella pertussis* antigen FHA were not detected in immunoproteomics studies, indicating that this technology may underrepresent the immunoreactive proteins in a pathogen. Although only one step in the pathway towards an efficacious approved vaccine, immunoproteomics is an important technology in the identification of novel vaccine antigens.

## INTRODUCTION

The value of vaccination as a means of combating life-threatening and chronic infectious diseases is well established. Many diseases which previously contributed to mortality are now prevented by vaccination. However, according to the WHO, about 1.5 million of deaths among children under five in 2008 were caused by vaccine preventable diseases. In addition many approved vaccines are more effective in young children than in adolescents and adults. Given that infectious diseases are still the leading global cause of morbidity and mortality and when considered in the context of both the rise in antimicrobial resistance and the lack of novel effective antibiotics in the drug development pipeline [1], the need for more efficacious vaccines for bacterial pathogens cannot be underestimated. 

There are several bacterial cell components incorporated into subunit vaccines, LPS, glycoconjugated vaccines, DNA vaccines, protein vaccines, which in addition to live attenuated vaccines and whole killed cell vaccines stimulate the host immune response in a variety of ways. Vaccine studies have often focussed on lipopolysaccharide (LPS) due to its potent stimulation of the immune response; however the antigenic diversity of the O-antigen component of LPS within species has limited the potential of LPS as potential vaccine antigens, for example *P. aeruginosa* LPS [2]. Many approved vaccines are glycoconjugate in nature (e.g. vaccines for *Neisseria meningitidis* and *Streptococcus pneumoniae*) and have proved effective against certain serogroups of pathogens; but they tend to suffer from the same issues with variability across strains. In addition, serotype switching and serotype replacement may undermine their effectiveness in the future [3]. Proteins are often more conserved across bacterial strains and have more potential to protect against several strains of the same bacterial species. 

Investigation and harnessing of the host-pathogen response are beneficial to two separate but essential aspects to vaccine development. Exploitation of the human humoral response by determining immunogenic bacterial protein using sera from vaccinated, colonised or recovering individuals has played an important part in identifying novel vaccine antigens. Identification of novel antigens is only part of the process of vaccine development, therefore examination of the host response in adults and in human volunteers at the cellular and humoral response level is also essential in the development of more efficacious vaccines. Protection against certain intracellular pathogens requires a strong cell-mediated immune response driven by polarisation towards a Th1 response; while other extracellular pathogens are best protected by a Th2 response which promotes antibody production and antibody-mediated cell killing [4]. Indeed knowledge of the interactions between pathogens and host cells *in vitro *is also critical, where the bacterial components that attach to host cells are investigated and elucidated. This review will focus on a number of examples of the exploitation of the host response in the identification of novel antigens and in the development of identified vaccine antigens as prophylactic therapies against respiratory bacterial diseases. 

## EXPLOITING THE MICROBIAL–HOST RESPONSE TO IDENTIFY NEW ANTIGENS

Reno Rappuoli and co-workers pioneered the use of a genomics approach to identify novel antigens against *Neisseria meningitidis *serogroup B using reverse vaccinology, in the late 1990s. They sequenced the genome of the pathogen, identified proteins which were likely to be surface associated, expressed these in *Escherichia coli* and administered the 350 proteins to mice to identify those that were immunogenic [5]. The sequencing step of the process took 18 months at the time. The development of genomics since then has meant a rapid expansion in the capabilities to identify new antigens. Over 2,800 bacterial genomes have been completely sequenced at time of writing, a figure which is increasing week on week (NCBI Microbial genomes: www.ncbi.nlm.nih.gov/genome). This vast bank of information combined with the advances in proteomics, including, the relatively reproducibility and ease of preparing 2-D gels has meant a rapid increase in proteomic analysis of bacterial pathogens which has given insights into mechanisms of bacterial virulence and pathogenesis, adaptation during chronic colonisation and host response. The natural step up to immunoproteomics, whereby 2-D blots are probed with host serum following infection or immunisation has greatly enhanced the identification of potential vaccine candidates, by enabling the discovery of novel proteins that stimulate the humoral host immune system. A key advantage of this approach is that final expressed bacterial proteins are used in the analysis, which have been completely processed and post-translationally modified by the pathogen. Several groups have used this method to discover potential vaccine candidates with varying degrees of success. Different sera have been used in these types of studies which range from using colonised patient sera, convalescent sera, or sera from challenged mice or rabbits. Furthermore, the majority of immunoproteomic studies have focussed on cell surface proteins or outer membrane proteins as this subgroup of bacterial proteins represent the interface for host pathogen interactions and regularly include adhesins. Other groups have examined secreted proteins as they are also primary antigen targets of the host immune response and include many virulence factors. Collectively, their exposure to the host immune system marks these pools of proteins as a rich source of vaccine candidates.

### Streptococcus Pneumoniae

* Streptococcus pneumoniae* is a Gram positive bacterium that is a leading cause of bacterial pneumonia, meningitis and pneumococcal septicaemia. It accounts for 20 to 50% of community acquired pneumonia cases and is the cause of over 820,000 deaths each year in children under five [6]. The seven-valent glycoconjugate pneumococcal vaccine Prevnar 7 has reduced the incidence of invasive pneumococcal disease in children and mortality from this disease; however, it is not optimal for protection in children under two or in immunocompromised patients [3]. Furthermore, there has been serotype replacement and increased incidence of non-vaccine serotypes, including the antimicrobially resistant serotype 19A, which now predominates in some countries [3]. Although the recently approved 10- and 13-valent vaccines target more serotypes and prevent invasive disease in children, there are at least 92 serotypes. This means that identification of protein antigens which are more highly conserved across the species than the capsular polysaccharides may present more effective alternatives. 

Immunoproteomics has been used to identify potential immunogenic antigens in *S. pneumoniae*. Adults develop antibodies to *S. pneumoniae* during asymptomatic carriage and it has been shown that the antibody response is age dependent [7,8]. This increase in antibody response to *S. pneumoniae* proteins which correlated with a reduction in morbidity in children prompted Ling *et al.*, to focus on antigens which showed increased immunogenicity with age of the child [9]. Two-dimensional Western blotting of cell wall fractions from *S. pneumoniae* probed with sera from healthy children ranging in age from 18 to 42 months or with sera from adults highlighted seventeen immunoreactive proteins. Two of these, both metabolic enzymes, were equally protective against two genetically different strains in a mouse challenge model. While this indicates promise for the proteomic approach to identify protective antigens, the protection obtained was only 36%. Another two antigens identified in this study, 6-phosphogluconate dehydrogenase (6-PGD) and Glutamyl-tRNA synthase were subsequently shown to be involved in adhesion to A549 cells [10,11]. While both independently protected mice against a lethal challenge, the level of protection was only marginally better than that observed with GAPDH and Fructose-bis-phosphate aldolase. In the case of 6-PGD, immunization also reduced lung and blood colonisation levels by several fold. The latter antigen, glutamyl-tRNA synthase shows promise due its lack of a human orthologue, the former protein has high homology to the human orthologue which could be detrimental to its development as a protective antigen [11]. 

A separate proteomic analysis of cell wall proteins identified proteins which were expressed across at least 40 *S. pneumoniae* serotypes and during animal infection [12]. Five proteins were evaluated in a mouse sepsis model, and although lung bacterial burdens were reduced following individual immunisations with two of the proteins, these did not prolong the survival of mice. The poorer outcome in this study, compared with that of Ling and co-workers, may be due to the fact that immunogenic proteins were not necessarily selected for this study. More recently, immunoproteomic analysis of the *S. pneumoniae *secretome also identified antigens which were shown to be immunogenic using immunised rabbit serum [13]. In total 54 proteins were detected, of which only 23 were identified, including several proteins which were known virulence factors, in addition to novel proteins. These have the potential as diagnostic markers in addition to vaccine candidates. Many of these immunogenic proteins had been previously identified in other species and in some cases by Ling *et al.*, e.g., Enolase, ZmpB, serine protease [9]. However, vaccine studies on these have not been reported to date. 

Overall, the comparable survival rates following lethal challenge (36 to 40%) for the four separate antigens described above and the lack of improved survival for others may suggest that more knowledge of the specific protective host response to *S. pneumoniae* is required to optimise further vaccine development. Indeed the finding that four distinct antigens showed very comparable survival rates may be a feature of the animal model used. It is possible that mice are not predictive of the immunological response to *S. pneumoniae* in humans. This is a key limitation of pre-clinical vaccination studies that must be always considered. An example of this is highlighted below in the discussion on *Bordetella pertussis* immunoproteomics studies. 

### *Burkholderia Pseudomallei* and other Members of Genus Burkholderia

*Burkholderia pseudomallei*, the causative agent of melioidosis, is a Gram-negative, intracellular pathogen endemic in Southeast Asia and Northern Australia. The disease is considerably variable in humans ranging from acute septicaemia, pneumonia to chronic or latent infection which can persist and emerge decades later. The bacterium is intrinsically antibiotic resistant and has a mortality rate up to 50%; however despite its significant morbidity and mortality, there is currently no licensed vaccine against this infection. Harding *et al.*, have used serum from convalescent patients to probe 2-D blots of *B. pseudomallei* OMPs [14]. By biotinylation of the bacteria prior to OMP preparation, they were able to focus solely on surface expressed proteins. They identified nine surface proteins which were immunoreactive, including elongation factor Tu (EF-Tu), polyribonucleotide nucleotidyl transferase and two chaperones, GroEL and DnaK. Among the biotinylated proteins identified were several that would be considered to be cytoplasmic. This highlights a common theme in proteomic analysis, that proteins predicted to be cytoplasmic and without any signal peptide are exposed at the bacterial surface [15]. A later study utilised rabbit serum to probe 2-D blots of membrane proteins and again identified elongation factor Tu (EF-Tu) and DnaK among the immunoreactive proteins of a less virulent related species, *B. thailandensis, *which shares 94% identity at the amino acid level with *B. pseudomallei *[16]. EF-Tu was shown to be secreted in outer membrane vesicles (OMVs) which are shed from *B. pseudomallei* during *in vitro* growth. Mucosal immunisation of mice with EF-Tu resulted in the generation of antigen specific IgG and IgA, polarised Th1 immune response and significantly reduced the bacterial burden in the lungs following a lethal challenge with *B. thailandensis*. OMVs are gaining momentum as a potential vaccine platform [17,18]. These are engineered proteoliposomes which incorporate the vaccine antigen, adjuvant in a particulate carrier. In a later study sub-cutaneous immunisation of mice with purified *B. pseudomallei* OMVs resulted in protection of 60% of mice against a lethal challenge with high OMV-specific titres, together with T-cell responses and humoral responses which were suggestive of a Th1 cell-mediated immunity [19]. It was not stated whether these purified OMVs contained EF-Tu or what their antigenic composition was. 

Immunoscreening of a lambda genomic library with pooled patient serum represents an alternative way of identifying immunoreactive antigens in *B. pseudomallei *[20]. This functional genomic approach allowed the screening of 5760 open reading frames which were then used to genetically immunise mice. Three clones were selected from an initial screen of 14 serum reactive phagemids, based on their strong reactivity. Serological evaluation of 74 patients showed that 94% of patients were seropositive for one of these proteins, OmpA*. B. pseudomallei* have several putative OmpA genes, cloning of these resulted in 6 recombinant proteins. Two of these were followed up, Omp3 which clustered with the OmpA of *E. coli* and *Klebsiella pneumonia *OmpA following multiple sequence alignment, and Omp7 which was closely related to OmpA of *Porphyromonas gingivalis *[20]. Subsequent mouse immunisation with these resulted in 50% protection against a lethal challenge for two different recombinant OmpA proteins with the most reactivity with sera from melioidosis patients [21]. When this pilot immunisation study is considered together with the widespread seropositivity for OmpA among convalescent melioidosis patients, it strongly suggest the potential for this antigen as one component of a future multi-valent vaccine against melioidosis. Using a similar approach, Su *et al.*, carried out a genomic survey of *B. pseudomallei*, screening an expression library with sera from melioidosis patients [22]. Among the 109 proteins identified from seropositive clones, one protein was selected, OMP85, for later study, as it is a highly conserved protein. Intraperitoneal immunisation of mice with this recombinant antigen resulted in 70% protection of mice over 15 days, as opposed to 10% in non-immunised mice in addition to significantly reduced bacterial load in lungs and spleen [23]. 

Most recently, protein microarrays have been used to identify immunoreactive proteins with plasma from recovered melioidosis patients or seropositive healthy individuals [24]. Of the 27 antigens identified, seven were more strongly recognised by plasma from recovered individuals over healthy controls. These represented antigens which were previously identified, and/or associated with virulence, including flagellin and flagellar hook associated protein, OmpA and BipB, confirming the capacity for patient serum to identify potential vaccine candidates. Although BipB was previously shown not to prolong survival of challenged mice [25], antibody levels against OmpA (one of the two OmpAs which was also found to be protective by Hara *et al.* [21]) were 10-fold higher in patients that had only one episode of melioidosis relative to those with recurrent disease, again indicating that antibodies specific to this protein may be protective. Another closely related pathogen, *Burkholderia cepacia* is one of 17 closely related species that comprise the *Burkholderia cepacia* complex [26]. This group of pathogens are responsible for chronic lung infections in people with cystic fibrosis and other immunocompromised individuals. The secretory proteins of *B. cepacia* were analysed on 2-D blots probed with serum from immunised mice [27]. One protein in particular showed a 96% homology with a metalloprotease from a *B. pseudomallei* strain and it was suggested that this could cross-protect against both pathogens. No *in vivo* challenge experiments were reported. 

Overall, investigation of immunoreactive proteins in *B. pseudomallei *using immunoproteomics or gene expression libraries has already allowed the identification of vaccine candidates to protect against melioidosis, and further identification of additional antigens should allow the development of a multivalent vaccine which will improve protection against *B. pseudomallei* and eliminate bacterial persistence. Taking both chronic infection and the intracellular lifestyle of *B. pseudomallei* into consideration, it is evident that there will be significant challenges in the development of an effective vaccine against this agent. A successful vaccine is likely to need the induction of both humoral and cell-mediated immunity for the complete protection against this pathogen. 

### Neisseria meningitidis

*N. meningitidis* colonises the upper respiratory tract, causing a mild respiratory infection, however in about 15% of patients it invades. There are five serogroups of this Gram negative pathogen which causes pneumonia, meningococcal meningitis and septicemia. Glycoconjugate vaccines are available which are effective against only four serogroups and it is widely recognised that the disease can be prevented by vaccination to induce antibody dependent complement mediated bactericidal activity [28]. No vaccine exists for serogroup B, which is responsible for over 30% of meningococcal disease in the US and up to 80% in Europe. In what was the first application of reverse vaccinology, several serogroup B antigens were identified which resulted in high titres of bactericidal antibodies, including, factor H binding protein (fHbp) and Neisserial heparin binding antigen (NHBA) [5]. Three highly immunogenic antigens (fHbp, Neisseria adhesin (NadA) and NHBA were combined with OMVs from an epidemic strain and are currently in phase III clinical trials. In contrast to the studies on other pathogens, a recent immunoproteomic analysis of *Neisseria meningitidis* failed to identify potentially protective antigens, i.e antigens which produced bactericidal antibodies when subsequently tested in mice [29]. Sera from both acute and convalescent patients were used to probe 2-D blots allowing the identification of 33 immunoreactive proteins. Interestingly many of the proteins identified were also shown to be immunogenic in other respiratory pathogens, e.g. GroEL, elongation factor-Tu, DnaK and cysteine synthase, but comparison of different patient sera indicated that meningococcal patients have very variable immune responses against a range of antigens. When the recombinant proteins were expressed and administered to mice, all sera had significant protein-specific ELISA titres, however none of these sera showed bactericidal activity. Protection of mice against challenge was not reported. 

### B. Pertussis

*Bordetella pertussis* is a Gram-negative bacterium responsible for the highly contagious respiratory infection, whooping cough (pertussis), in humans. Pertussis vaccination exemplifies the benefits of vaccination and the issues that arise when vaccine uptake wanes. The whole cell pertussis vaccine had a major impact on reducing childhood mortality when it was introduced in the 1940s, however, serious side effects, including encephalitis, in a subgroup of vaccinated children led to its reduce uptake and consequent widespread outbreaks throughout the 1970. Introduction of the acellular pertussis vaccine improved confidence and consequently uptake of the pertussis vaccination with the effect of reducing the incidence among children for the next decade. However, while vaccination decreased the incidence of this infection, there are reports of a resurgence of pertussis among adults and adolescents in recent years, in developed countries, despite good uptake of pertussis vaccination [30]. The current 5-component acellular vaccine administered in combination with diphtheria and tetanus toxoids (DTaP) and also combined with *Haemophilus influenzae* B and polio antigens, contains five *Bd. pertussis* antigens. The antigens include pertussis toxoid, filamentous hemagglutinin (FHA), fimbriae 2 and 3 and pertactin. These antigens are all virulence factors and play a role in bacterial attachment (reviewed by Marzouqi *et al.* [31], and antibodies to these have been identified in infected patients [32]. There is renewed interest in improving pertussis vaccination which has led to a number of immunoproteomics studies. These, however, highlight some limitations of immunoproteomics. Two separate studies of *Bd. pertussis* surface proteins [33] and secretome [34] were carried out using mouse antisera from inactivated *Bd. pertussis* immunised mice as the primary antibody. The surface immunoproteomes for two strains of *Bd. pertussis* were nearly identical. Among the 27 immunoreactive proteins identified across both strains, pertactin which is a component of the current approved pertussis vaccine, was identified. Interestingly, although FHA is present in the surface proteome and is a highly immunogenic component of acellular pertussis vaccines, it was not identified as an immunogenic protein among both lab strains examined in this study [33]. This may be due to its very large molecular size and consequent poor transfer to blotting membranes. Another study of immunoreactive proteins in *Bd. pertussis* analysed the serum of children that had been immunised with the whole cell pertussis vaccine also identified pertactin among the immunoreactive proteins, but neither FHA nor pertussis toxin. Zhu *et al.*, compared the murine immunoproteome with the human immunoproteome of *Bd. pertussis* and found considerable differences in immunoreactivity [35]. Only four human immunoreactive proteins were detected from immunised or infected mice and many human immunodominant antigens were not recognised by murine immune sera. This clearly demonstrates that bacterial antigens may be recognised differently by the murine and human immune systems and may indicate that researchers should be cautious when using murine antisera, or indeed antisera from other species, in the identification of potential human vaccine antigens. 

## INVESTIGATING THE HOST RESPONSE TO ENHANCE THE EFFICACY OF IDENTIFIED ANTIGENS: DEVELOPMENT OF A *P. AERUGINOSA* VACCINE

The identification of vaccine antigens by immunoproteomics or other immunomic analysis, although powerful technologies, is only the first step in a long process to successful protective vaccine. Indeed, several immunogenic proteins identified have failed to protect against challenge in subsequent animal models (for example, BipB in *B. pseudomallei *[25] and GroEL and EF-Tu in *N. meningitidis* [29]. A full knowledge of the host response that is required for a protective response in humans is essential in the development of an effective vaccine. The process involved is exemplified by the separate development of vaccine candidates against *P. aeruginosa*. This is an opportunistic pathogen which colonises 80% of adults with the genetically inherited disease cystic fibrosis, contributing to the mortality and morbidity of that disease. It is also responsible for a significant proportion of ventilator associated pneumonias and colonises and invades burn patients causing sepsis. *P. aeruginosa* strains are characterised by intrinsic resistance to a range of antimicrobial agents and antibiotics and have multiple mechanisms of resistance which means that treatment is difficult. In susceptible populations, prevention of infection would represent a better approach to therapy and indeed, prevention of infection with *P. aeruginosa* is a major objective in the treatment of chronic obstructive pulmonary disease and cystic fibrosis. The development of protective vaccines to protect susceptible populations against this pathogen spans several decades. The majority of the work has focussed on a limited number of antigens that showed promise early in their development. The potential for outer membrane protein OprF was first identified as a protective antigen as early as 1984 [36] and its suitability confirmed when it was demonstrated using a panel of monoclonal antibodies as being a conserved protein, with several conserved surface epitopes [37]. It was evaluated as a hybrid protein with another conserved outer membrane protein OprI, which was also antigenically cross-reactive against seventeen serogroups [38]. This recombinant hybrid protein was subsequently shown to protect immunosuppressed mice against *P. aeruginosa* challenge and was shown to be tolerated in human volunteers [39]. Antibodies against OprF have been shown to be protective in burn and ocular models of *P. aeruginosa* infection [40,41], however, only partial responses have been achieved in cystic fibrosis patients, so the recent focus of development of this vaccine antigen has been in understanding the host response and optimising the adjuvant/ vaccine administered to maximise the protective response. Investigation of the host response in chronically colonised CF patients has suggested that a Th2 response correlated with infection, while a Th1 cell response correlated with an improved outcome and may be more protective [42,43]. Bumann *et al.*, have shown that a live vaccine constructed from a number of attenuated *Salmonella *vaccine strains induced mucosal responses in human volunteers when administered orally or nasally [44]. This phase I/II clinical trial on the OprF-OprI conjugate vaccine demonstrated high levels of specific antibodies, including IgA at the bronchial surface and showed that the nasal route was superior in terms of mucosal antibody response [44]. While this work towards an OprF-OprI vaccine to protect children with CF against *P. aeruginosa *is promising, a Phase III randomised control trial will be necessary to determine its efficacy and to demonstrate that antibody titres in this case, correspond to protection. An alternative immunisation approach using adoptive transfer of OprF-pulsed dendritic cells as the vaccine in a mouse model showed significant protection and clearance of *P. aeruginosa* and activation of a Th1 response with a concomitant reduction in inflammatory cell recruitment. They also observed protection against a mucoid strain which is generally associated with chronic colonisation of the CF lung, albeit a slightly delayed response relative to the non-mucoid strain [45].

Understanding the host pathogen interaction of these vaccines will allow the development of future vaccines which protect against different pathogens. Extensive host response studies have been carried out on the proteins involved in this OprF-OprI hybrid antigen. OprF has been shown to involved in attachment of *P. aeruginosa* to host cells [46] and is upregulated during chronic infection, in contrast to other virulence factors which are either lost or down regulated [47]. OprF is involved a range of other virulence characteristics in *P. aeruginosa*, including Type III secretion system, production of virulence factors such as pyocyanin, elastase and exotoxin A and adhesins [46], again illustrating the potential of virulence factors and adhesins as protective antigens. It has been suggested that OprF is a host immune system sensor enabling *P. aeruginosa* to enhance virulence when bacteria are in contact with the host [46]. 

Independently, another aspect of the *P. aeruginosa* attachment has been exploited, leading to the development of flagellin and flagella-based vaccines which have been tested in Phase III clinical trials. Most CF patients immunised developed high serum titres to flagella A and B and a significant reduction in the number of immunised patients that developed *P. aeruginosa* infection was observed among the group of patients that received all four vaccinations [48]. Disappointingly, the study did not show a significant reduction in chronic infection in these patients, or an alteration in the rate of decline in lung function. In a follow-on analysis of the immune response to vaccination, Campodonico *et al.*, showed that the polymeric flagella rather than monomeric flagellin appeared to be superior for generating immunity to *P. aeruginosa*. Antibodies to type a and b flagella were more potent in mediating opsonic killing of *P. aeruginosa *and mediating passive immunity in mice than serum raised to flagellin. This was attributed to flagellin inducing high titres of antibodies which could neutralise the innate immunity due to TLR5 activation [49].

Combinations of both outer membrane proteins OprF and OprI and flagellins have also been evaluated in a multimeric fusion protein vaccine, in order to further optimise the protective host response by exploiting the ability of flagellin to activate TLR-5 and act as an adjuvant as well as an antigen [50]. Immunisation of mice with a combination of an OprF epitope fused with OprI incorporated at the N-terminus of the type A or type B flagellin resulted in high level of antibodies to OprF, flagellin and OprI. The Opr/flagellin recombinant proteins elicited high titres of IgG2a, in addition to IgG1, although the response was biased towards a Th1 response. In addition, immunisation reduced the bacterial burden in mice challenged with non-mucoid *P. aeruginosa* strains. One limitation of this study was that although antigen specific antibodies were able to activate complement, complement-mediated killing was only effective against non-mucoid bacteria, being less effective against mucoid bacteria. The conversion of *P. aeruginosa* to a mucoid phenotype during chronic infection which may have significant impacts on the exposure and expression of surface antigens and is likely to be an additional challenge in developing effective vaccines to protect against this pathogen. 

There are considerable advances yet to be made both in our knowledge of the host response and in the development of an effective vaccine for this difficult pathogen, again demonstrating that antigen identification is only a fraction of the process to a successful vaccine. These studies also highlight the limitations of immunoproteomics to predict good vaccine antigens. Although flagellin type B protein (FliC) was identified in an immunoproteomics analysis of extracellular *P. aeruginosa *proteins using CF patient serum [51], confirming its potential to produce high antibody titres; neither OprF nor OprI were identified in this study. The authors attributed the presence of flagellin and other OMPs among extracellular immunogens to bacterial cell lysis. However, OprF and OprI would also be expected to be present in a bacterial lysate. Their lack of detection in this study could suggest that they were not released from the cell surface, under the conditions tested, or like certain *Bd. pertussis* antigens these may not be amenable to detection by immunoproteomics. 

## CONCLUSIONS

Examples of immunoreactive proteins identified by immunoproteomics in a range of respiratory pathogens are listed in (Table **1**). Many of these are common across several unrelated bacteria. For example, GroEL (HSP60) and DnaK (HSP70), are both conserved heat shock proteins and are highly immunoreactive in several diverse pathogens including *Shigella flexineri*, *Helicobacter pylori* and *Yersinia entercolitica *[52-54]. Immunisation with GroEL, but not DnaK resulted in 100% protection in mice following *Bacillus anthracis* challenge [55]. GroEL immunisation was also protective against *S. pneumoniae *[56]. EF-Tu is another antigen which is immunoreactive across several pathogens. Interestingly, while it was protective against *B. thailandensis*, it was not protective when used in immunisation studies followed by challenge with *N. meningitidis*. In addition, it was among the immunoreactive proteins that were identified with serum from both successfully and unsuccessfully vaccinated mice using an attenuated *Francisella tularensis* vaccine strain, suggesting that antibodies against EF-Tu are not protective against this pathogen [65]. EF-Tu has been shown to bind to host proteins such as fibronectin, mucin and plasminogen [57-59] and as a result may play a direct role in the microbial host response for certain pathogens. This may be an important criterion in discerning the most protective antigens among all those identified in an immunoproteomics study. Immunoreactogenicity needs to be considered alongside the potential pathogenicity of any individual protein, as protective vaccine antigens tend to be those that play a role in pathogenesis. Many immunoreactive proteins identified in the studies outlined here are metabolic enzymes and would be considered to have a cytoplasmic localisation within the cell. The fact that they are immunogenic during infection in animals and patients, could be due to bacterial cell lysis or could be due to these enzymes having as yet an undetermined role in bacterial virulence and are worthy of further study. 

In summary, the advances in immunoproteomics have led to a massive increase in the potential vaccine candidates which may be useful to protect against difficult to treat bacterial pathogens. While this review has focussed on respiratory pathogens, many immunoreactive antigens have also been identified using this approach for other bacterial pathogens, e.g. *Staphylococcus epidermidis *[60], *Campylobacter concisus *[61]. As outlined, this potent technology is not without limitations, but when used with serum from either colonised or convalescent patients, it is particularly useful as it allows researchers to identify immunoreactive antigens that are expressed during human infection. Further thorough examination of the host immune response, including immunisation studies in a number of carefully selected models, will help identify which of the immunogenic proteins can be taken further to human trials. It is likely that multivalent vaccines can be developed by careful selection of immunoreactive antigens identified with immunomic technologies and when combined with the appropriate adjuvant will increase our weaponry in the defence against respiratory bacterial infections. 

## Figures and Tables

**Table 1.** Examples of Immunoproteomics Studies to Identify Vaccine Antigens and/or Virulence Factors in Respiratory Pathogenic Bacteria

Pathogen	#	Antigen(s) of interest	Serum source	Protein fraction	Protective	Comment	Ref
*Bd. pertussis*	30	Pertactin, serum resistance protein (BrkA), OmpP, sulphate binding protein (Sbp)	Immunised children (whole cell pertussis vaccine)	Total membrane proteins & extracellular proteins	NA	Observed differences between mouse and human serum data	[35]
*Bd. pertussis*	1	Peptidoglycan associated lipoprotein	Immunised mice	Cell lysate	NA	immunodominant antigen	[62]
*Bd. pertussis*	25	Serum resistance protein, pertactin, HSP-60, HSP70,Clp protease, serine protease, EF-Tu, Glutamyl-tRNA aminotransferase, phosphoenolpyruvate synthase	Immunised mice	Soluble proteins	NA	Comparison of two vaccine strains	[34]
*Bd. pertussis*	11	Serum resistance protein, pertactin, serotype fimbral subunit, HSP-60, HSP-10, ATP synthase	Immunised mice	Surface proteins	NA	Comparison of two vaccine strains	[33]
*B. cepacia*	18	EF-Tu, pyruvate carboxylase, ATP-dependent zinc metalloprotease, tyrosyl-tRNA synthase, phospholipid glycerol acyltransferase, ABC efflux pump	Immunised mice	Secreted proteins	NA		[27]
*B. pseudomallei*	12	GroEL, DnaK, PhaP, Ef-Tu, polyribonucleotide nucleotidyltransferase	Immunised rabbit serum	Surface proteins	NA	9 Antigens both biotinylated and immunoreactive	[14]
*B. thailandensis*	16	Ef-Tu, DnaK, AhpC, oxodipate CoA succinyltransferase, HSP10,	Rabbit from B. mallei immunised rabbits		Protection	EF-Tu immunoprotective	[63]
*F. tularensis*	31	F1F0 ATP synthase, EF-Tu, glutamylt-RNA synthase, fructose bisphosphatealdolase, GAPDH, pyruvate dehydrogenase, DnaK	Tularemia patients and vaccinated individuals		NA		[64]
*F. tularensis*	9	EF-Tu, GroEL, ATP synthase, DNA gyrase, chitinase family 18 protein, DNA directed RNA polymerase	Mouse sera from Live attenuated immunised mice	Soluble and membrane enriched	NA	Comparison of responses from successful vaccination and unsuccessful vaccination – examples of successful vaccination in bold.	[65]
*Leptospira interrogans*		Lipoprotein L21, L41, L32. Loa22.	Rat infection	Membrane proteins, *in vitro* culture and guinea pig infection	NA		[66]
*P. aeruginosa*	51	PasP protease, zinc dependent aminopeptidase, azurin, OprH, LasB protease, PrpL protease	CF patient sera	Extracellular	NA	Compared 2 strains, PA01 and PA14	[51]
*N. meningitidis*	33	TufA (EF-Tu), alcohol dehydrogenase, DnaK, cysteine synthase, aconitasehydratase, ATP synthase F1, Opa900	Convalescent patients	Total protein	No serum bactericidal antibodies produced		[29]
*S. pneumoniae*	17	Glutamyl-tRNAamidotransferase,GAPDH, fructose bis-phosphate aldolase, 6-PGD	Healthy children and adults (asymptomatic carriage)	Surface proteins	4 tested, all showed protection		[9-11]
*S. pneumoniae*	23	Enolase, serine protease, N-acetylglucosamine-6-phosphate deacetylase, general stress protein, pneumococcal histidine triad protein D precursor , Zinc metalloprotease	Immunised rabbits	Secreted proteins	No *in vivo* studies	All 6 listed immunoreactive proteins found in all 3 clinical strains tested.	[13]

# Number of antigens identified.
